# Age-specific *Plasmodium* parasite profile in pre and post ITN intervention period at a highland site in western Kenya

**DOI:** 10.1186/s12936-017-2119-y

**Published:** 2017-11-16

**Authors:** Ednah N. Ototo, Guofa Zhou, Lucy Kamau, Jenard P. Mbugi, Christine L. Wanjala, Maxwell Machani, Harrysone Atieli, Andrew K. Githeko, Guiyun Yan

**Affiliations:** 1 Centre for Global Health Research, Kenya Medical Research Institute (KEMRI), PO Box 1578, Kisumu, 40100 Kenya; 2 Kenyatta University, PO Box 43844, Nairobi, Kenya; 3 Masinde Muliro University of Science and Technology, PO Box190-50100 Kakamega, Kenya; 4 Program in Public Health, University of California, Irvine, CA 92697 USA

## Abstract

**Background:**

Monitoring and evaluation of entomological, parasitological and clinical data is an important component of malaria control as it is a measure of the success of the interventions. In many studies, clinical data has been used to monitor trends in malaria morbidity and mortality. This study was conducted to demonstrate age dependent prevalence of malaria in the pre- and post-interventions period.

**Methods:**

A series of cross-sectional malaria parasitological surveys were conducted in Iguhu, western Kenya. Participants were randomly selected school-aged children between 6 and 13 years. The study was conducted between June 2002–December 2003 and January 2012–February 2015. Sexual and asexual parasite prevalence and densities were determined using microscopy. Age-dependence in parasite infections was compared between 2002–2003 and 2012–2015.

**Results:**

*Plasmodium falciparum* had the highest prevalence of 43.5 and 11.5% in the pre- and post-intervention periods. *Plasmodium malariae* had a prevalence of 2.3 and 0.2%, while *Plasmodium ovale* had a prevalence of 0.3 and 0.1% during the pre- and post-intervention period, respectively. There was a 73.7% reduction in prevalence of *P. falciparum* in the post-intervention compared to the pre-intervention period. *Plasmodium falciparum* parasite density increased by 71.2% between pre- and post-intervention period from (geometric mean of) 554.4–949.2 parasites/µl. Geometric mean gametocytaemia in Iguhu was higher in the post-intervention period (106.4 parasites/µl), when compared to the pre-intervention period (54.1 parasites/µl). Prevalence and density of *P. falciparum* showed a lower age-dependency during post-intervention period when compared to pre-intervention period.

**Conclusion:**

The study provides evidence for reduction of malaria prevalence following the introduction of LLINs and ACT in western Kenya. Fewer people become infected but the few infected may be more infectious as suggested by higher gametocyte densities. The high parasite densities, which were not dependent on age, observed in the post intervention period imply that a more comprehensive integrated malaria management may be required to sustain the current interventions and hence reduce malaria transmission.

## Background

Monitoring and evaluation of entomological, parasitological and clinical data is important to measure effectiveness of malaria control programmes. Most commonly, clinical data has been used to monitor trends in malaria morbidity and mortality. Active surveillance involving longitudinal cohort studies are used in obtaining parasitological data. While hospital-based passive surveillance captures only symptomatic cases, a field based active surveillance system captures trends in both symptomatic and asymptomatic cases and is thus less biased.

The major malaria intervention strategies in Kenya include vector control using indoor residual spraying (IRS) and long-lasting insecticide-treated nets (LLINs) [1–3], and early treatment and diagnosis. In 2006, the Government of Kenya initiated distribution of subsidized insecticide-treated nets [4], followed by a universal distribution of the LLINs in 2011 with a goal of one net for at least two people. LLIN distributions target vulnerable groups including women and children after successful randomized trials showed a reduction in malaria morbidity and mortality [5]. Kenya has adopted artemisinin-based combination therapy (ACT) as first-line for the treatment of uncomplicated malaria since 2004 due to widespread resistance to chloroquine and sulfadoxine–pyrimethamine (SP) [6, 7]. These interventions have been reported to reduce the global mortality due to malaria by 48% [8, 9].

A reduction in transmission can result in altered or reduced immune acquisition which would increase the population vulnerability to unstable transmission, epidemics and severe disease [10]. An age specific parasite profile is important as a monitoring tool to show the trend of malaria transmission in order to advise where the control efforts should focus among different age groups. To demonstrate the age groups affected by malaria, a study was conducted on age-specific *Plasmodium* parasite profile during pre- and post-intervention periods in western Kenya.

## Methods

### Study design and study area

This study is based on a long-term Plasmodium parasitological surveillance programme starting in June 2002 to date. Snapshots of this data was taken between June 2002 and December 2003, and January 2012–February 2015, this being the pre- and post-intervention period. The data in the two time periods was aggregated. The relative change in parasite profile was determined in log transformed nonlinear data.

**Fig. 1:**
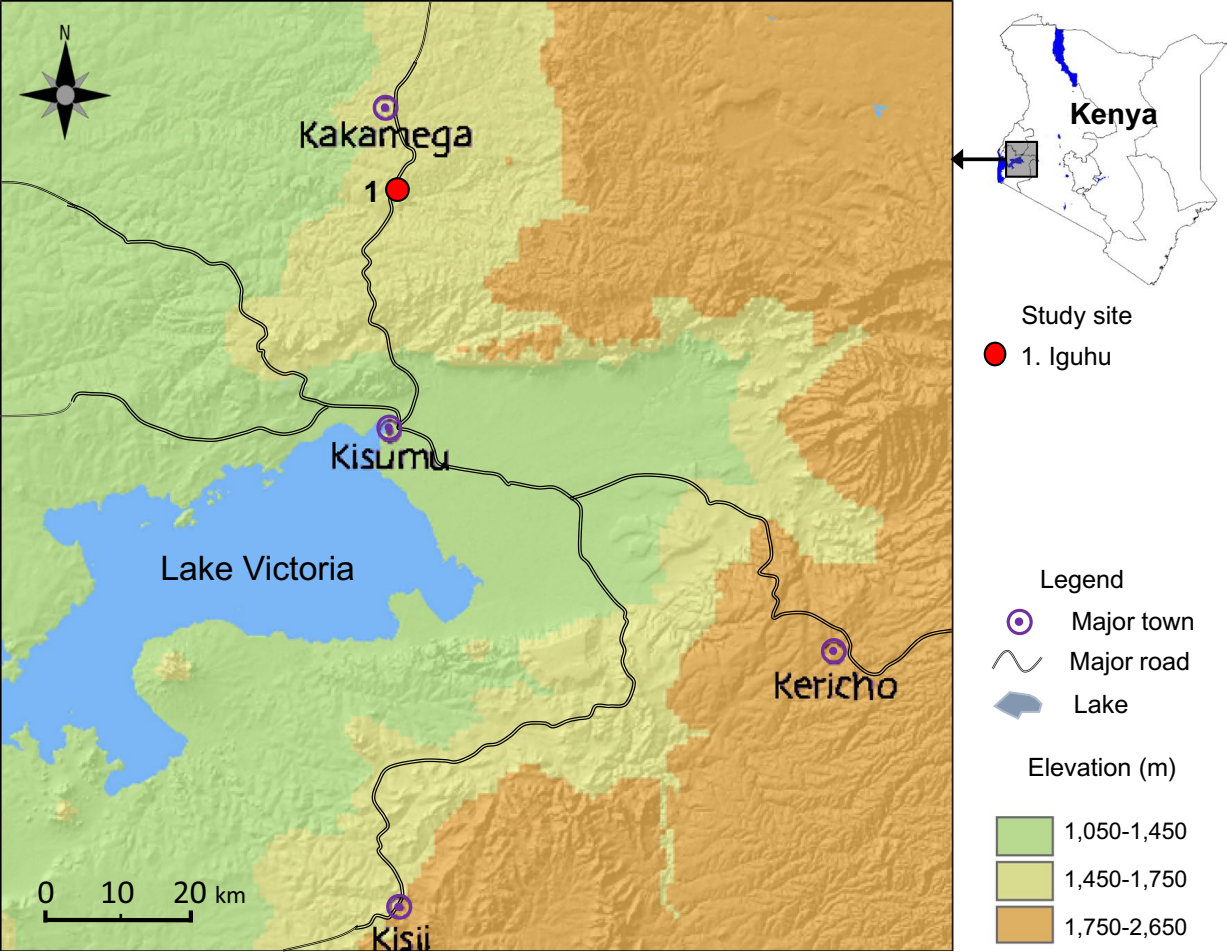
Map showing the study site in the western Kenya. Iguhu is a highland site that has mesoendemic malaria transmission

The study was conducted in Iguhu, western Kenya (1430–1580 m above sea level) (Fig. 1). The area is characterized by year-round malaria transmission with a peak during the rainy season in May. Climate in western Kenya consists mainly of two seasons of rainfall, a long rainy season between March and May and a short one between October and November [11]. The topography of the region is a main factor influencing malaria transmission. The highland region has flat valleys that create breeding habitats for mosquito during the rainy season [12].

### Parasitological survey

A series of cross-sectional malaria parasitological surveys were conducted between June 2002–December 2003 and January 2012–February 2015. Volunteer school-aged children, age between 6 and 13 years, were randomly selected during each survey. Among those children who assented and had consents from their guardians, blood samples were drawn using finger-prick method. The sample size was calculated based on the size of study population and parasite prevalence from a previous study [11]. In each survey, finger-prick blood samples were collected, and thick and thin smears were prepared for malaria parasite species identification and parasite counts using microscopy. Malaria parasite counts were read against 200 white blood cells and density was expressed as parasites per ml assuming a count of 8000 white blood cells per ml [11]. Two readings were made per slide by two trained microscopists, and 20% of the slides were randomly selected for further verification by senior external microscopists for quality control.

### Statistical analysis

*Plasmodium* parasite/gametocyte prevalence was expressed as the ratio of positive samples over the total number of samples tested. χ^2^-test was applied to compare parasite/gametocyte prevalence between the two survey periods i.e. 2002–2003 and 2012–2015. Age-specific blood parasite density of *Plasmodium falciparum* for each site was log-transformed to stabilize variance. Geometric mean parasite/gametocyte densities were compared for the two survey periods by *t* test assuming unequal variance for geometric means. Age-dependence was analysed using regression analysis for parasite/gametocyte prevalence and density, R^2^ was calculated and significance tested.

## Results

### *Plasmodium* parasite profiles

A total of 8325 blood slides were collected and examined in the study sites (Table 1). *Plasmodium falciparum* had the highest prevalence 43.5 and 11.5% in the pre- and post-intervention periods. *Plasmodium malariae* had a prevalence of 2.3 and 0.2% while *Plasmodium ovale* had 0.3 and 0.1% during the pre and post intervention period, respectively (Table 1). The average age of children surveyed were very similar during the two surveys, with mean ± SD of 9.6 ± 2.2 years in 2002–2003 and 9.8 ± 1.4 years in 2012–2015. Whereas, overall parasite prevalence, prevalence of *P. falciparum* parasite and gametocyte, and prevalence of *P. malariae* parasite were significantly lower during 2012–2015 surveys compare to 2002–2003 surveys (χ^2^-test, P < 0.05) (Table 1).

**Table 1:** *Plasmodium* parasite profile in Iguhu

	2002–2003		2012–2015	
Parameters	Sample size	Prevalence (%)^a^ (95% CI)	Sample size	Prevalence (%)^a^ (95% CI)
Total	4753		3572	
*P. falciparum* (Pf)	2068	43.5 [42.1, 44.9] a	411	11.5 [10.5, 12.6] b
*Pf* gametocytes (Pfg)	131	2.8 [2.3, 3.2] a	46	1.3 [0.9, 1.7] b
*P. malariae* (Pm)	110	2.3 [1.9, 2.7] a	6	0.2 [0.03, 0.3] b
*P. ovale* (Po)	16	0.3 [0.2, 0.5] a	5	0.1 [0.02, 0.3] a
Pf + pm	80	1.7 [1.3, 2.0] a	2	0.1 [0, 0.1] b
Pf + Po	8	0.2 [0.1, 0.3] a	2	0.1 [0, 0.1] a
Pf + Pm + Po	2	0.04 [0, 0.1]	–	–
Pm + Po	0	–	–	–
N. positive	2150	45.2 [43.8, 46.6] a	424	11.9 [10.8, 12.9] b

^a^Prevalence in the same row were significantly different if they are connected by different letters (χ^2^-test, P = 0.05)

There was a 73.7% reduction in prevalence of *P. falciparum* in the post intervention period (45.2% vs. 11.9%, χ^2^ = 1062.9, d.f. = 1, *P* < 0.0001) compared to the pre-intervention period (Table 2). Whereas the *P. falciparum* density increased by 71.2% between pre- and post-intervention period from (geometric mean of) 554.4–949.2 parasites/µl (t = 6.05, d.f. = 548, two-tail P < 0.0001) (Table 2).

**Table 2:** Parasitaemia and gametocytaemia profile

Parameters^a^	Year		P-value^b^ (2-sided)
	2002–2003	2012–2015	
GM parasitaemia	554.4	949.2	< 0.0001
25% value	200	280	
50% value	560	800	
75% value	1560	2600	
GM gametocytemia	54.1	106.4	< 0.001
25% value	40	40	
50% value	40	80	
75% value	80	200	

^a^GM: geometric mean

^b^t-test assuming unequal variance for geometric means

### Gametocyte prevalence and densities

Gametocyte prevalence was observed to decrease from 2.8% in the pre-intervention period to 1.3% in the post intervention period (χ^2^ = 21.1, d.f. = 1, *P* < 0.0001) (Table 2). Geometric mean gametocytaemia in Iguhu was higher in the post intervention period (106.4 parasites/µl) when compared to the pre-intervention period (54.1 parasites/µl) (t = 3.62, d.f. = 53, two-tail P < 0.001) (Table 2).

### Age-dependency of *Plasmodium falciparum* infections

**Fig. 2:**
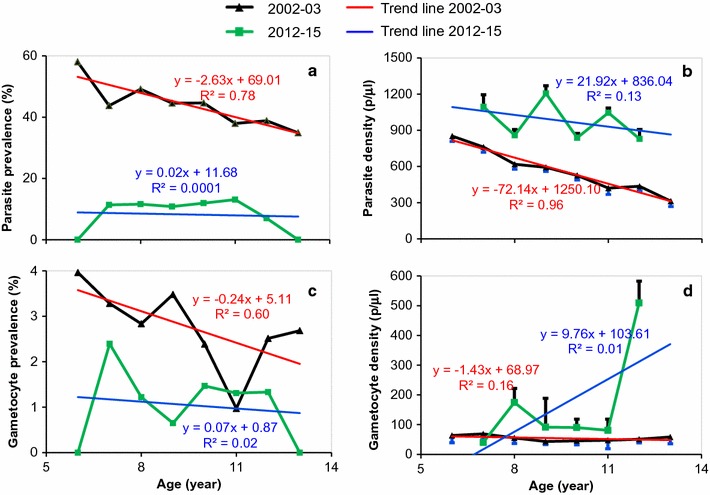
**a**. *Plasmodium falciparum* prevalence in relation to age; **b** *P. falciparum* parasite density (geometric mean) in relation to age; **c** *P. falciparum* gametocyte prevalence in relation to age; **d** *P. falciparum* gametocyte density (geometric mean) in relation to age between the years 2002–2003 and 2012–2015. Error bars in mean density represent standard error

Prevalence and density of *P. falciparum* showed a lower age-dependency during post-intervention period when compared to pre-intervention period (Fig. 2). Parasite prevalence and density showed a significant negative correlation with age in the pre-intervention period (R^2^ = 0.78 and R^2^ = 0. 96), however it did not show any age-dependence in the post intervention period (R^2^ = 0.0001 and R^2^ = 0.13) (Fig. 2a, c). Similar result was found for gametocyte prevalence (R^2^ = 0.60) during the pre-intervention period and (R^2^ = 0.01) in the post intervention period (Fig. 2b, d).

## Discussion

Findings from this study indicate that there was relatively high prevalence of *P. falciparum* before the introduction of the use of insecticide-treated bed nets in 2006 and a reduction of prevalence post-intervention. During the pre-intervention period, there was also a reported resistance in the drug of choice to treat the malaria parasite, SP [6]. Following the roll-out of LLINs and ACT in 2004, malaria prevalence showed a reduction—indicating the efficacy of these control measures. In line with findings from the current study, previous studies have shown declining trend of malaria following introduction of LLINs and ACT [13]. Bed nets ownership increased from 7% in the pre-intervention period [14] to 80% in the post-intervention period in Iguhu [15].

The introduction of the interventions in the study site reduced exposure to the parasite hence the prevalence was reduced. However, in 2015 despite the low parasite prevalence across the study sites, parasite densities doubled. This clearly shows a reduced ability to suppress parasitaemias. A small reservoir of parasitaemias may sustain transmission in a community due to their high infectiousness [16]. Individual Infections have been reported to persist for up to 13 years [17].

The presence of *P. falciparum* gametocytes in asymptomatic carriers increases the infectiousness of humans to mosquitoes as they are not likely to seek medical treatment and serve as reservoirs for continuous transmission of the parasite [18]. The use of ACT for treatment of uncomplicated malaria clears gametocytes and reduces the number of individuals acting as reservoirs for transmission [19, 20]. In the current study, the prevalence of gametocytes was higher during intervention period than pre-intervention period. Gametocyte infections can be sub-microscopic [18], and hence can be missed especially when using microscopy. Ongoing studies in the same sites are investigating the presence of sub-microscopic sexual and asexual parasites. The gradual increase in gametocyte prevalence shows that parasite suppression has not yet occurred. It has been shown in western Kenya that the infectiousness of an individual to malaria vectors is inversely related to the duration of exposure to malaria transmission [16]. As asymptomatic individuals do not seek treatment, integrated vector management can be used so as to block transmission of the gametocytes to the vectors.

Parasitaemia is proportional to the duration of exposure to malaria transmission. Thus, older individuals have lower parasitaemia compared to younger individuals such as children in the same environment [12, 20]. The present study showed that while parasite densities declined with age during pre-intervention period, higher parasite densities were seen in older children during intervention period.

There is a limitation with the accuracy of parasite densities. In this study, standard WHO procedures were used to measure parasite densities. However, due to the limitation of human ability, parasite density estimates may be with errors. For example, if microscopists look longer and more carefully, they may count more sexual and/or asexual parasites. Double readings method which allowed per slide to be read by two trained microscopists was used, and further quality control, where 20% of the slides were randomly selected for further verification by senior external microscopists. These measures minimized the parasite counting errors. In the future, quantitative PCR may be used to more accurately estimate parasite density.

## Conclusions

The study provides evidence of reduction of malaria prevalence following the introduction of LLINs and ACT in western Kenya. Fewer people become infected but the few infected may be more infectious as suggested by higher gametocyte densities. The high parasite densities observed, which were not dependent on age, imply that a more comprehensive integrated malaria management may be required to sustain the current interventions and hence reduce malaria prevalence. This may include both additional vector control measures such as larviciding, and parasite transmission control, such as judicious use of single low dose primaquine to reduce gametocyte in the highlands where transmission is low. It may also involve identification of malaria transmission foci that can be targeted with additional interventions such as larviciding and IRS.

Continuous monitoring of the parasitological profile is recommended to detect further changes in asexual and sexual parasite prevalence and density so as to inform malaria control programme on what additional steps need to be taken to further reduce the disease prevalence. In general, reduction in malaria transmission and disease prevalence may result in reduced functional immunity and increase the vulnerability of the population to severe disease in case interventions are interrupted. The method described here of using age-related parasite densities provide a simple, accurate, reliable and reproducible field technique for indicating important changes in population based parasitological profiles. The development of functional immune assays would greatly help in providing information on trends in immune status of populations with reduced exposure to malaria. This data would justify the continued use of entomological interventions even as the disease moves towards elimination and eradication.
